# NVP-BKM120 potentiates apoptosis in tumor necrosis factor-related apoptosis-inducing ligand-resistant glioma cell lines via upregulation of Noxa and death receptor 5

**DOI:** 10.3892/ijo.2015.3035

**Published:** 2015-06-05

**Authors:** KIMBERLY A. FOSTER, ESTHER P. JANE, DANIEL R. PREMKUMAR, ALEJANDRO MORALES, IAN F. POLLACK

**Affiliations:** 1 Department of Neurosurgery, Pittsburgh, PA, USA; 2 University of Pittsburgh School of Medicine, Pittsburgh, PA, USA; 3 University of Pittsburgh Cancer Institute Brain Tumor Center, Pittsburgh, PA, USA

**Keywords:** glioma, BKM120, TRAIL, apoptosis, Noxa

## Abstract

We previously observed that glioma cells are differentially sensitive to TRAIL-induced toxicity. Based on our observation that TRAIL-resistant glioma cell lines typically exhibited high levels of Akt activation, we hypothesized that inhibition of Akt signaling using the PI3 kinase inhibitor NVP-BKM120 could promote TRAIL-induced apoptosis in gliomas. We assessed this combination in established and primary cultured glioma cells. Combination treatment led to significant cellular death when compared to either drug alone, but had no effect in normal human astrocytes, and demonstrated activation of the caspase cascade. This enhanced apoptosis appears dependent upon the loss of mitochondrial membrane potential and the release of Smac/DIABLO, AIF and cytochrome c into the cytosol. The upregulation of Noxa and sequestration of Mcl-1 by Noxa were important factors for cell death. Knockdown of Noxa abrogated apoptosis and suggested dependency on Noxa in combination-induced apoptosis. BKM120 upregulated cell surface expression of death receptor 5 (DR5), but did not increase levels of the other major TRAIL receptor, death receptor 4 (DR4). This study demonstrates that antagonizing apoptosis-resistance pathways, such as the PI3/Akt pathway, in combination with death receptor activation, may induce cell death in TRAIL-resistant glioma.

## Introduction

Malignant glioma is an aggressive brain tumor that responds poorly to conventional treatment modalities (1–3). Gliomas demonstrate multiple modes of resistance and single-agent therapy has proven ineffective in treatment of malignant glioma (4,5). While the underlying basis for resistance to apoptosis is not fully understood (6,7), recent discoveries as to the pathogenesis of glioblastoma have led to the development of rationally-designed, targeted therapies aimed at disrupting key signaling pathways.

Tumor necrosis factor-related apoptosis inducing ligand (TRAIL), a member of the TNF family, is a potential cancer agent due to its tumor-specific induction of apoptosis and human recombinant TRAIL is being tested in clinical trials for various cancer types (8–12). TRAIL initiates apoptosis by binding one of two main receptors, death receptor 4 and 5 (DR4 and DR5, respectively), which signal via an adaptor intermediate molecule that promotes formation of a death-inducing signaling cascade (DISC), and ultimately activation of caspase-8 and caspase-3 (13). While most human cancer cell lines express death receptors for Apo2L/TRAIL, many cancer types have proven resistant to TRAIL-induced cellular death (14–16). We, among others, have shown that many malignant gliomas are also TRAIL resistant, despite expression of TRAIL receptors (17–19) and aberrance in the NF-κB, protein kinase C, Bcl-2 and Akt pathways have all been implicated in TRAIL resistance in glioma (19–21). In particular, Akt pathway activation, which can occur secondary to PTEN deletion and/or PIK3CA mutations, is common in malignant glioma (22).

**Figure 1:**
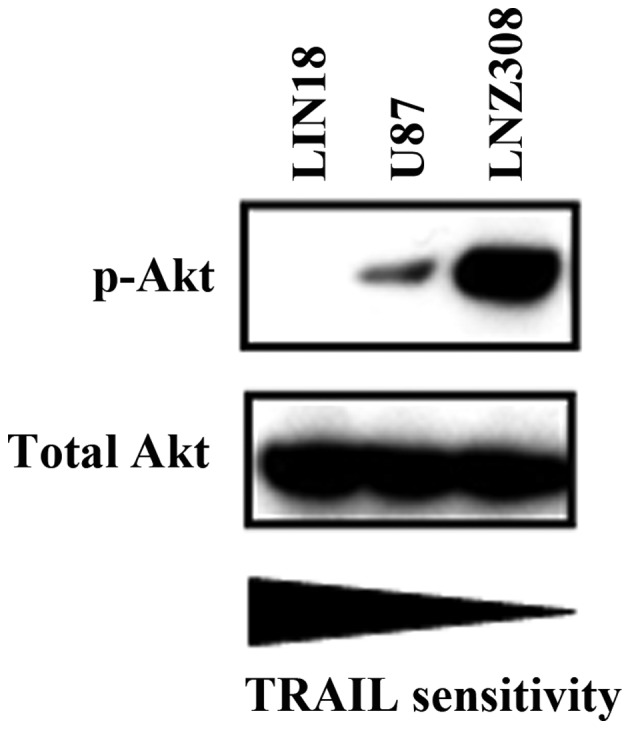
TRAIL-resistant glioma cell lines exhibit high levels of Akt activation. Untreated LN18, LNZ308 and U87 cell lines were examined and cell extracts were subjected to western blot analysis with the indicated antibody.

NVP-BKM120 is a highly selective pan-class I phosphatidylinositol-3 kinase (PI3K) inhibitor (23), shown to effectively induce apoptosis in tumor cell lines and animal models of cancer at clinically achievable doses (24–26), and a preliminary clinical trial suggests this agent is well-tolerated (27). A recent study demonstrated effective, synergistic induction of apoptosis in lung cancer cell lines when cells were co-treated with BKM-120 and TRAIL (28). To date, it has not been reported whether BKM120 can sensitize TRAIL-resistant malignant glioma cell lines to apoptosis. We previously observed that TRAIL-resistant glioma cell lines often exhibited high levels of Akt activation (Fig. 1) and, thus, we hypothesized that Akt pathway inhibition via the PI3 kinase inhibitor BKM120 could work with TRAIL to promote cell death in human glioma.

Herein, we show the combination of BKM120 and TRAIL in glioblastoma is effective in promoting cellular death and demonstrate that the effectiveness of this combination is likely contingent on the upregulation of the pro-apoptotic protein Noxa and the death receptor DR5.

## Materials and methods

### Cell lines

Two TRAIL-resistant cell lines were utilized (U87 and LNZ308). U87 was obtained from American Type Culture Collection (Manasas, VA, USA) and LNZ308 was kindly provided by Dr Nicolas de Tribolet (Switzerland). We obtained primary GBM cells from Conversant Biologics (Huntsville, AL, USA). Normal human astrocytes (HA) and media were obtained from ScienCell Research Laboratories (Carlsbad, CA, USA). Cell culture conditions of these cell lines were as previously described (29,30).

### Reagents and antibodies

Soluble human recombinant SuperKillerTRAIL (referred to as TRAIL in this study) was purchased from Enzo Biochemicals (Enzo Life Sciences, Farmingdale, NY, USA). NVP-BKM120 was purchased from Chemie Tek (Indianapolis, IN, USA). Caspase inhibitors (z-VAD-fmk, z-IETD-fmk, z-DEVD-fmk, and z-LEHD-fmk) were purchased from R&D Systems (Minneapolis, MN, USA). The following antibodies were used: Mcl-1 (#4572), cytochrome c (#4280), cleaved poly-ADP-ribose polymerase (PARP, #9546), cleaved caspase-3 (#9664), cleaved caspase-8 (#9496), cleaved caspase-9 (#9501), Smac/DIABLO (#2954), pAKT (#5106), AKT (#2920), pS6kinase (#5364), pBAD (#9105), BIM (#2819), Bcl-2 (#2872), Bcl-xL (#2764), surviving (#2808), XIAP (#2042), DcR1 (#4756) and β-actin (#4970) were from Cell Signaling Technology (Beverly, MA, USA). Noxa (sc-26917) and apoptosis-inducing factor (AIF; sc-5586) were from Santa Cruz Biotechnology (Santa Cruz, CA, USA). DR4 (#IMG-141A) and DR5 (#IMG-122A) were from Imgenex (San Diego, CA, USA).

### Annexin V apoptosis assay

Apoptosis was evaluated using a fluorescent Annexin V/propidium iodide (PI) assay kit (Invitrogen, Carlsbad, CA, USA) as described previously (29,30). Cells were treated with or without inhibitors for various intervals, harvested, and pelleted by centrifugation (1,000 rpm for 5 min); washed in ice-cold phosphate-buffered saline (PBS); and re-suspended in the Annexin V-fluorescein isothiocyanate/PI reagent in the dark for 15 min before flow cytometric analysis using a FACSCalibur flow cytometer (BD Biosciences, San Jose, CA, USA).

### DiOC6 labeling and detection of mitochondrial membrane depolarization

Mitochondrial membrane depolarization was measured as described previously (29,30). Nonadherent cells were collected, and attached cells were trypsinized and resuspended in phosphate-buffered saline (PBS). Cells were loaded with 50 nM 3′,3′-dihexyloxacarbo-cyanine iodide (DiOC6; Invitrogen), which accumulates in intact mitochondria, at 37°C for 15 min. Cells were then spun at 3,000 × g, rinsed with PBS, and resuspended. Fluorescence intensity was detected by flow cytometry and analyzed with CellQuest (BD Biosciences) and FlowJo (Tree Star, Inc., Ashland, OR, USA) analysis software.

### Immunoprecipitation and western blot analysis

Western blot and immunoprecipitation were performed as previously described (18). Cells were washed in ice-cold PBS and lysed in buffer containing 30 mM HEPES, 10% glycerol, 1% Triton X-100, 100 mM NaCl, 10 mM MgCl_2_, 5 mM EDTA, 2 mM Na_3_VO_4_, 2 mM β-glycerophosphate, 1 mM phenylmethyl-sulfonyl fluoride, 1 mM 4-(2-aminoethyl) benzenesulfonyl fluoride, 0.8 μM aprotinin, 50 μM bestatin, 15 μM E-64, 20 μM leupeptin, and 10 μM pepstatin A for 15 min on ice. Samples were centrifuged at 12,000 × g for 15 min, supernatants were isolated, and protein was quantified using protein assay reagent (Pierce Chemical, Rockford, IL, USA). Equal amounts of protein were separated by SDS-PAGE and electro-transferred onto a nylon membrane (Invitrogen). Nonspecific antibody binding was blocked by incubation of the membranes with 4% bovine serum albumin in Tris-buffered saline (TBS)/Tween-20 (0.1%). The membranes were incubated with primary antibody overnight at 4°C, washed in TBS/Tween-20, and incubated with a 1:2000 dilution of horseradish peroxidase-conjugated secondary antibody in TBS/Tween-20 at room temperature for 1 h. Proteins were visualized by western blot chemiluminescence reagent (Cell Signaling). Where indicated, the membranes were re-probed with antibodies against β-actin to ensure equal loading and transfer of proteins.

For immunoprecipitation, cell extracts were prepared by lysing 5×10^6^ cells on ice for 30 min in CHAPS lysis buffer (10 mM HEPES, pH 7.4, 150 mM NaCl, 1% CHAPS, protease, phosphatase inhibitors). Lysates were clarified by centrifugation, and equal amounts of protein extracts were incubated overnight with primary antibody. Dynabeads Protein G (Invitrogen) was added for 2 h, followed by magnetic separation of the immunoprecipitated fraction; western blot analysis was conducted as outlined already herein. Scanning densitometry was performed using acquisition into Adobe Photoshop (Adobe Systems, Inc., San Jose, CA, USA) followed by image analysis (Un-Scan-It gel, version 6.1; Silk Scientific, Orem, UT, USA).

### Subcellular fractionation

Cells were treated with or without inhibitors, and cytosolic proteins were fractionated as described previously (29). Cells were resuspended in lysis buffer containing 0.025% digitonin, sucrose (250 mM), HEPES (20 mM, pH 7.4), magnesium chloride (5 mM), potassium chloride (10 mM), EDTA (1 mM), phenylmethylsulfonyl fluoride (1 mM), 10 μg/ml aprotinin, and 10 μg/ml leupeptin. After 10 min of incubation at 4°C, cells were centrifuged (2 min at 13,000 × g), and the supernatant (cytosolic fraction) was removed and frozen at −80°C for subsequent use.

### Transient transfection

Transient transfection was performed as previously described (18). Optimal 29mer-pRS-small hairpin (sh) RNA constructs were obtained from Origene (Rockville, MD). Sequences specific for human Noxa (GGA GGT GCT ACA CAA TGT GGC GTC GGC AC), Mcl-1 (ACC TAG AAG GTG GCA TCA GGA ATG TGC TG) and control sequences (GCA CTA CCA GAG CTA ACT CAG ATA GTA CT) (non-target shRNA) were used for this study. Glioma cells were seeded in six-well plates and allowed to reach 70% confluence. Transfection of targeting or control shRNA was performed using FuGENE6 per the manufacturer’s recommendations (Roche Applied Science, Indianapolis, IN, USA). One microgram of Mcl-1 or Noxa or nontargeting shRNA in 100 μl of Opti-MEM medium was mixed with 2 μl of FuGENE6 and incubated at room temperature for 20 min, followed by the addition of complete medium to make the total volume up to 2 ml. After 48 h, media were changed and cells were incubated with inhibitors or vehicle for 24 h. Assessment of cell viability (Annexin V binding) was carried out as described (18).

### Flow cytometry of death receptors

Cells were analyzed for surface expression of DR4 and DR5 by indirect staining with anti-human DR4 and DR5 (Imgenex), followed by FITC-conjugated IgG. Briefly, cells (1×10^6^) were stained with 300 μl PBS containing saturating amounts of anti-DR4 and anti-DR5 overnight at 4°C. After incubation, cells were washed with PBS and subjected to secondary antibody (IgG) for 1 h at room temperature. After washing, expression of death receptors were subjected to flow cytometric analysis using a FACSCalibur flow cytometer (BD Biosciences).

### Statistical analysis

Unless otherwise indicated, data are expressed as mean ± SD for at least three separate experiments performed in triplicate. The significance of differences between experimental conditions was determined using a two-tailed Student’s t-test. Differences were considered significant at P-values ≤0.05.

## Results

### Combination treatment with BKM120 and TRAIL induces apoptosis in TRAIL-resistant glioma lines but not human astrocytes

**Figure 2:**
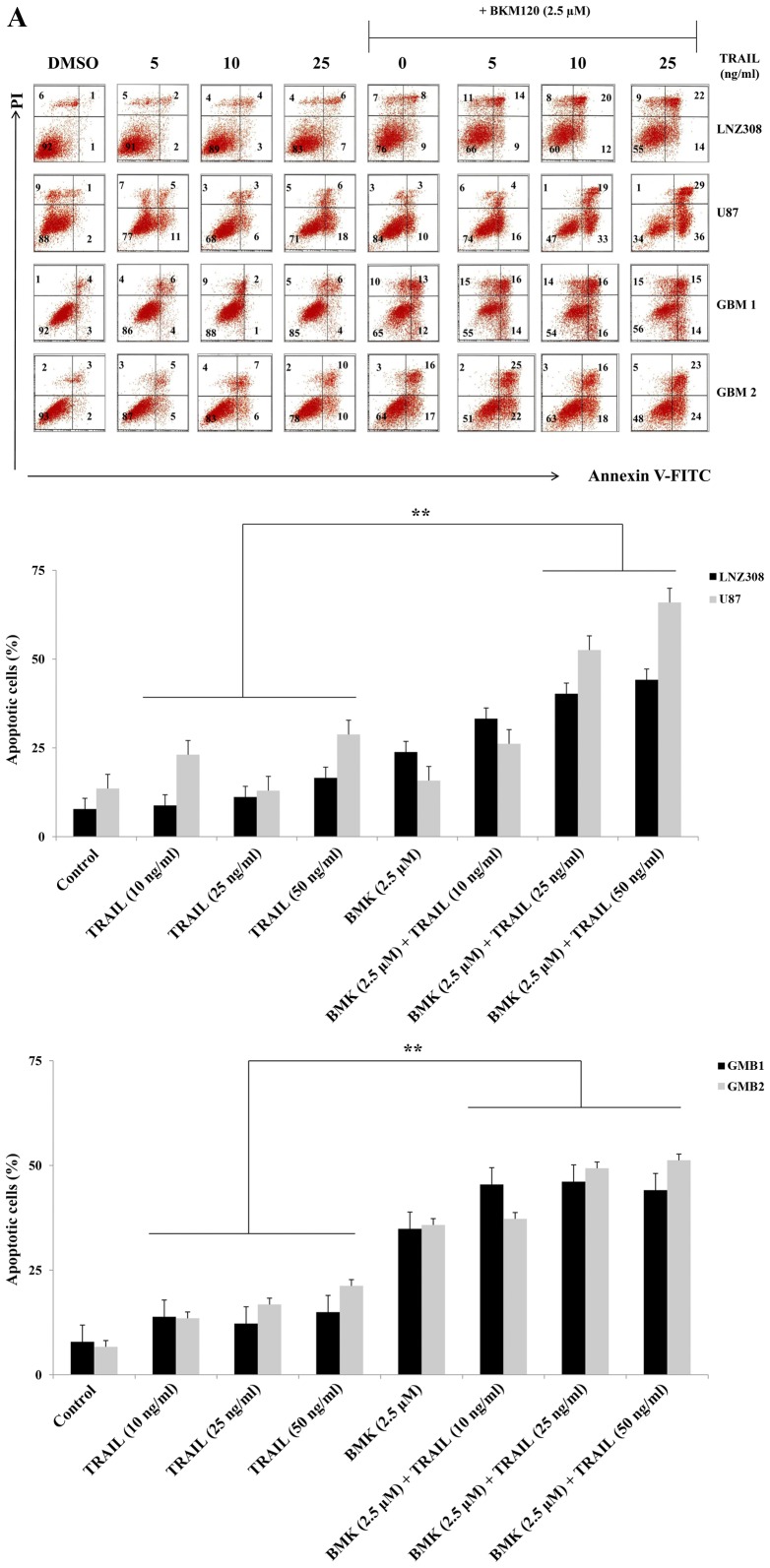

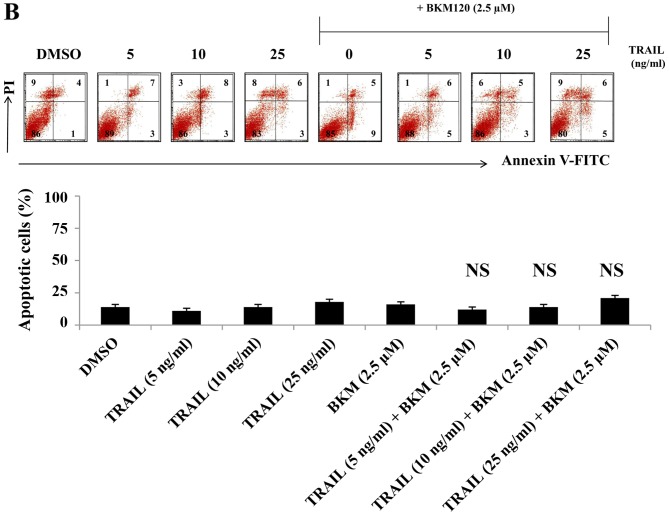
Combination treatment of BKM120 and TRAIL induces apoptosis in TRAIL-resistant glioma lines but not human astrocytes. (A) LNZ308 and U87 cell lines and primary cultures established from 2 glioblastoma (GBM) patient samples (GBM 1 and GBM 2) were treated as shown for 24 h and frequency of apoptotic cells was determined by cell surface Annexin V binding assay. The histograms are representative of one of at least three different experiments. Bar graphs are representative of three independent experiments. Standard deviation was <5% and error bars are shown. ^**^p<0.05. (B) Normal human astrocytes were exposed to BKM120 or TRAIL or the combination of both for 24 h and frequency of apoptotic cells was determined by Annexin V binding assay. Standard deviation was <5% and error bars are shown. NS, no statistically significant difference between treatment and control.

To quantitatively assess the effects of this combination, LNZ308 and U87 cells were subjected to an Annexin V/PI analysis. Single agent treatment resulted in modest Annexin labeling, while co-treatment enhanced Annexin V/PI positive labeling. To demonstrate that sensitivity to this combination was not restricted to established cell lines, primary cultures were created from patients diagnosed with glioblastoma and were modestly sensitive to BKM120 or TRAIL as single agents, while the combination induced apoptosis (Fig. 2A). Statistical significance was reached when comparing single agent TRAIL treatment to combination treatment for both the established and primary lines. Human astrocytes were subjected to individual agents and co-treatment for 24 h and analyzed by Annexin V/PI assay; neither individual agents nor co-treatment showed significant toxicity in non-neoplastic human astrocytes (Fig. 2B). Having validated the hypothesis that inhibition of Akt signaling via BKM120 can promote TRAIL activity, we examined potential mechanisms involved, including promotion of caspase cleavage and activation of the mitochondrial apoptotic pathway.

### Co-treatment of TRAIL-resistant glioma cell lines with BKM120 and TRAIL activates the caspase cascade

**Figure 3:**
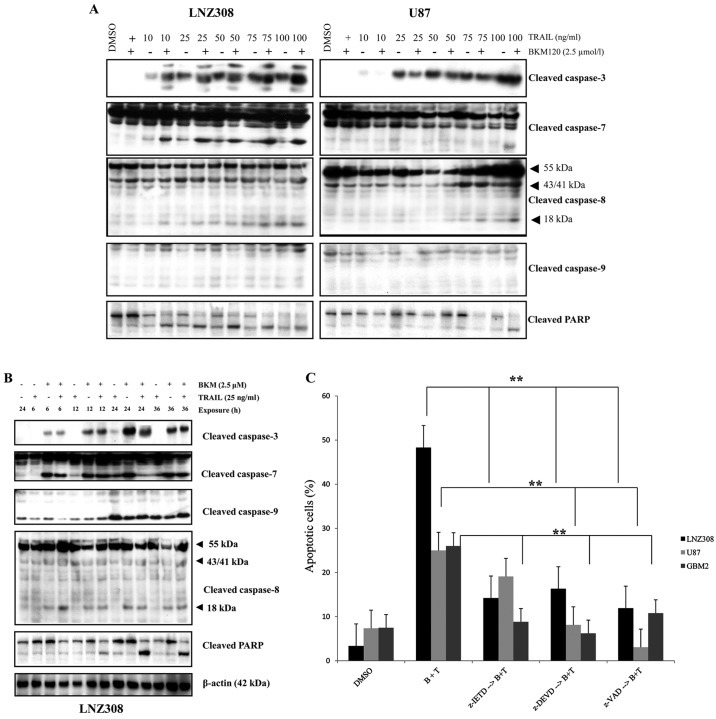
Cotreatment of TRAIL-resistant glioma cell lines with BKM120 and TRAIL activates the caspase cascade. (A) LNZ308 and U87 cell lines were treated with TRAIL or BKM120 or the combination of both at designated doses for 24 h. Cell extracts were subjected to western blot analysis with indicated antibody. Arrows correlate to the molecular weights indicated in parentheses next to the name of protein and β-actin served as the loading control. (B) LNZ308 and U87 cells were treated with TRAIL or BKM120 or the combination of both for 6, 12, 24 and 36 h. Cell extracts were subjected to western blot analysis with indicated antibody. Arrows correlate to the molecular weights indicated in parentheses following the name of protein and β-actin served as loading control. (C) LNZ308 and U87 cell lines and primary patient sample (GBM 2) were pre-incubated with caspase-specific inhibitors for 2 h (z-DEVD-fmk, caspase-3 inhibitor; z-IETD-fmk, caspase-8 inhibitor; z-VAD-fmk, pan-caspase inhibitor) and the effect on cell death was examined with Annexin V/PI assay. Bar graph is representative of three independent experiments. Standard deviation was <5% and error bars are shown. ^**^p<0.05.

Western blot analysis [dose-response (Fig. 3A) and time-course (Fig. 3B)] demonstrates activation of the main effector caspase-3 more robustly with combination treatment than with treatment of TRAIL or BKM120 alone and in a time-dependent manner, as early as 6 h in LNZ308; cleavage of PARP was seen as early as 12 h. The initiator caspase-8 was activated by proteolytic cleavage as shown by intermediate (43-kDa) and active (18-kDa) fragments. The downstream caspase-7 showed early cleavage in LNZ308.

As caspases appeared to have a central role in BKM120 and TRAIL-mediated apoptosis, LNZ308, U87 and primary patient GBM cells were preincubated with caspase-specific inhibitors (z-DEVD-fmk, caspase-3 inhibitor; z-IETD-fmk, caspase-8 inhibitor; z-VAD-fmk, pan-caspase inhibitor) and the effect on cell death was examined with Annexin V/PI assay. Inhibition of caspase-3 and use of a pan-caspase inhibitor decreased co-treatment-induced cellular death in all lines, while caspase-8 inhibition significantly diminished cell death in LNZ308 and the primary patient sample (Fig. 3C).

### BKM120 and TRAIL co-treatment induces loss of mitochondrial membrane potential (Δψm) and release of mitochondrial protein into the cytosol

**Figure 4:**
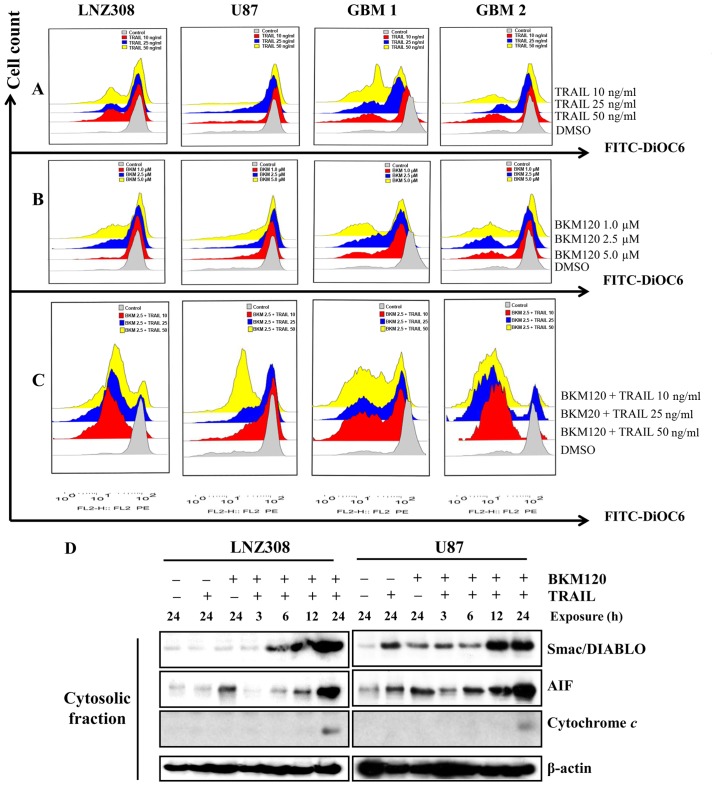
BKM120 and TRAIL co-treatment induces loss of mitochondrial membrane potential (Δψm) and release of mitochondrial protein into the cytosol. Established glioma cells (LNZ308 and LN229) and primary cultures from 2 GBM patients (GBM 1 and GBM 2) were treated with the indicated concentrations of TRAIL (A), BKM120 (B) or the combination of both (with BKM120 dose of 2.5 μM) (C) for 18 h. The integrity of the mitochondrial membranes of the cells was examined by DiOC6 staining and flow cytometry. Decrease in fluorescence intensity reflected loss of Δψm. Data are representative of three independent experiments (D) LNZ308 and U87 cells were treated with TRAIL or BKM120 or the combination of both for the indicated duration. Cytosolic extract was prepared, and equal amounts of protein were separated by SDS-PAGE and subjected to western blot analysis with the indicated antibodies. β-actin served as loading control.

Mitochondria play the central role in the intrinsic pathway of cellular death. Evidence suggests that loss of mitochondrial Δψm and release of soluble proteins, such as cytochrome c, into the cytosol are major events associated with intrinsic pathway apoptosis (30,31). To evaluate if mitochondrial-related cell death played a role in combination-induced apoptosis, we utilized the DiOC6 assay. In both established and primary cells, TRAIL (Fig. 4A) and BKM120 (Fig. 4B) induced minimal loss in mitochondrial Δψm, while co-treatment significantly increased the cellular population with loss of mitochondrial Δψm (Fig. 4C). This result was most robustly seen in LNZ308 and the primary patient samples.

Loss of mitochondrial Δψm is associated with release of mitochondrial intermembrane proteins into the cytosol that are critical in activating initiator caspases. We evaluated the release of Smac/DIABLO, apoptosis-inducing factor (AIF) and cytochrome c and observed accumulation in the cytosolic fraction (Fig. 4D) after combination exposure. The release of Smac/DIABLO and AIF occurred as early as 6 h post-treatment in both lines, while cytochrome c showed expression at 24 h.

### BKM120-induced Noxa upregulation and Noxa interaction with Mcl-1 may potentiate TRAIL toxicity

**Figure 5:**
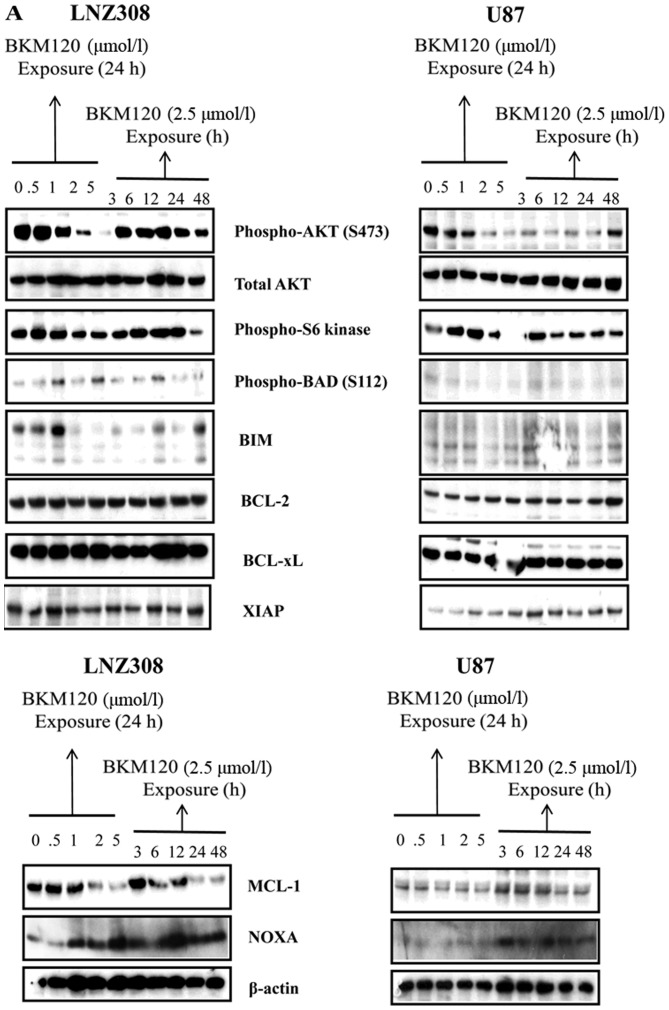

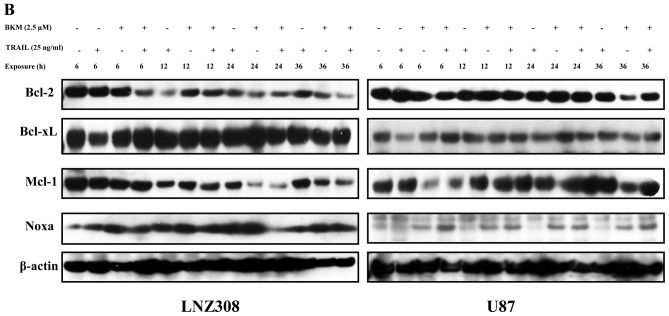

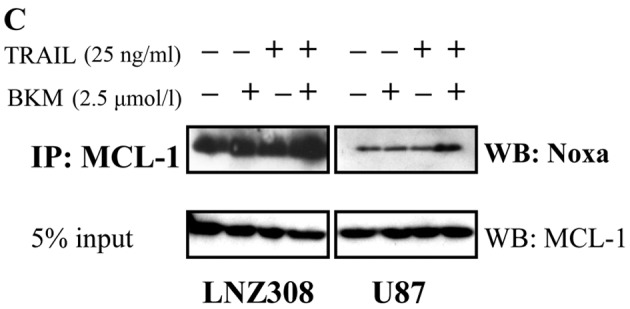

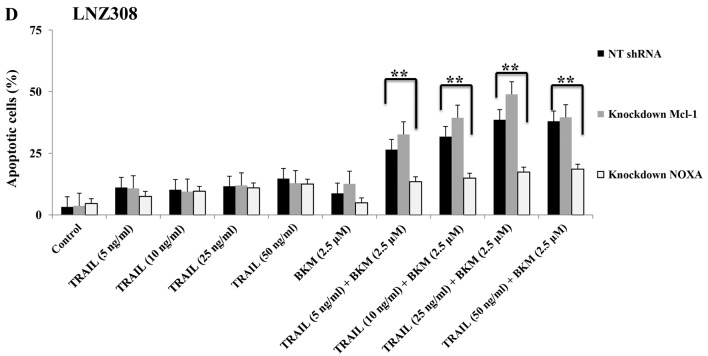
BKM120-induced Noxa upregulation and Noxa interaction with Mcl-1 may potentiate TRAIL toxicity. (A) Cells were treated with BKM120 at indicated doses for 24 h, as well as exposed to BKM120 2.5 μmol/l for indicated time and examined in a time-dependent manner. Cell extracts were subjected to western blot analysis with indicated antibody. β-actin served as loading control. (B) LNZ308 and U87 cells were treated with indicated concentrations of TRAIL or BKM120 or combination of both for indicated duration. Cell extracts were subjected to western blot analysis with indicated antibody. β-actin served as loading control. (C) LNZ308 and U87 cells were treated with indicated concentrations of TRAIL or BKM120 or combination of both for 24 h. An equal amount of protein (500 μg) was immunoprecipitated (IP) with Mcl-1 antibody and subjected to western blot analysis using the indicated antibodies. IP:Mcl-1 probed with Mcl-1 served as control (IP:Mcl-1; WB:Mcl-1). (D) LNZ308 cells were subjected to non-target (control), Noxa and Mcl-1 shRNA, as described in Materials and methods. Following transfection, cells were incubated in the presence of SAHA and ABT-737 (indicated concentration) for 24 h. Control cells received DMSO (vehicle). At the end of the treatment period, the viable cell numbers were determined by flow cytometric analysis. The mean number of apoptotic cells acquired from two independent experiments is shown. The standard deviation was <5% and error bars are shown. ^**^p<0.05 values were considered statistically significant and represent the decrease in percent of apoptosis following transfection with shNoxa; shMcl-1 transfection showed a trend towards increased apoptosis compared to control (NT shRNA), but was not statistically significant.

Levels of the Bcl-2 family members Mcl-1 (anti-apoptotic) and Noxa (pro-apoptotic), as well as their interaction, play a critical role in determining TRAIL lethality (18,20,32–34). Therefore, we hypothesized that shifting Bcl family protein homeostasis may contribute to BKM120-TRAIL cellular death. Western blot analysis demonstrated that target inhibition occurred with BKM120 exposure, as levels of pAKT (but not total AKT) were diminished in both lines in a time- and dose-dependent manner (Fig. 5A). We examined whether BKM120 exposure could alter the levels of proteins known to be crucial to both the intrinsic and extrinsic apoptotic pathways, including Bcl-2 and XIAP family members.

We found little effect on expression of major Bcl-2 family proteins Bcl-2 and Bcl-xL or the IAP family member XIAP (Fig. 5A); however, we observed increased levels of Noxa, a pro-apoptotic Bcl-2 family member, and decreased levels of Mcl-1, a well-established pro-survival protein and downstream target of the Akt-signaling pathway, in a time- and dose-dependent manner (Fig. 5A). Levels of survivin, phosphorylated Bad (S112) and BIM remained unchanged (data not shown).

Next we examined expression levels in a time-dependent manner after co-treatment with TRAIL and BKM120 and again found downregulation of Mcl-1 and upregulation of Noxa, with unchanged levels of Bcl-2 and Bcl-xL (Fig. 5B). To determine if this combination alters Mcl-1:Noxa interactions, given that sequestration of the pro-survival Mcl-1 by Noxa could tip the balance to cellular death, we performed immunoprecipitation of Mcl-1 and subjected the product to western blot analysis of Noxa. The combination increased Mcl-1:Noxa association (Fig. 5C). To examine the hypothesis that Mcl-1 downregulation and Noxa upregulation are required for TRAIL-induced apoptosis, we performed RNA interference experiments to knock-down the expression of both proteins. We transiently transfected and treated cells as indicated in Fig. 5D, then analyzed cellular viability with flow cytometry. After co-treatment, a decrease in Noxa levels resulted in a statistically significant decrease in cell toxicity, while Mcl-1 knockdown showed a trend toward increased death after co-treatment.

### BKM120 upregulates cell surface expression of DR5

**Figure 6:**
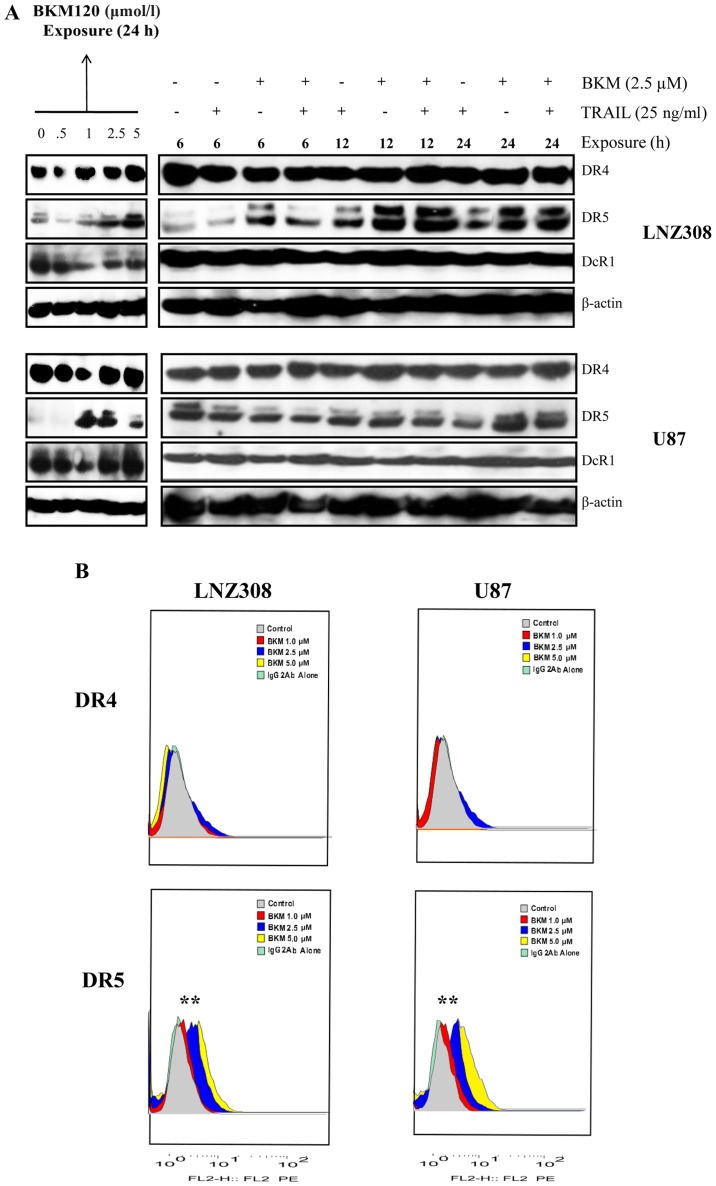
BKM120 upregulates cell surface expression of DR5. (A) Cells were treated with BKM120 at indicated doses for 24 h, as well as exposed to TRAIL (25 ng/ml) or BKM120 (2.5 μM) or combination for the indicated time and examined in a time-dependent manner. Cell extracts were subjected to western blot analysis with the indicated antibody. β-actin served as loading control. (B) Cells were treated with BKM120 in mono-treatment at indicated doses for 24 h. Cell surface expression of death receptors was analyzed by flow cytometry. Images are representative of data from 3 independent experiments. ^**^p<0.05 values considered statistically significant, representing the difference between control and BKM120 at 2.5 μM.

The death receptor-induced apoptotic pathway (also called the extrinsic apoptotic pathway) is initiated by death ligands, most commonly Fas ligand (FasL), tumor necrosis factor (TNF), and TRAIL. It has been shown that Akt inhibition upregulates FasL (35,36) and that PI3K inhibition can sensitize glioblastoma to death receptor-induced apoptosis (3); we hypothesized that Akt inhibition would affect expression of death receptors. In our study, BMK120 treatment produced an increase in the expression of DR5, with evidence of protein expression at 1 μmol/l at 24 h post-treatment. There was no change in the expression levels of DR4 or decoy receptor 1 (DcR1) in either cell line (Fig. 6A). In a time-dependent manner, BKM120 alone and combination treatment produced increased levels of DR5 in both lines as early as 6 h, but no time-dependent change in expression of DR4 or DcR1 was observed. FACS analysis showed that the cell surface expression of DR5 was increased in LNZ308 and U87 cell lines treated with BKM120, without a change in the expression of DR4 (Fig. 6B).

## Discussion

Drug resistance remains a major barrier for cancer therapeutics and multiple pathways contribute to cell death resistance in glioma. Currently, there is great interest in aberrant PI3K/Akt signaling in glioma and increased activity of this pathway may be responsible for the dismal prognosis associated with glioblastoma (37). While many human cancer cells express death receptors, various cancer types, including some glioma cell lines, remain resistant to TRAIL. Akt pathway activation, a frequent finding in human glioma, correlates with TRAIL resistance. Studies have shown that inhibition of PI3K/Akt pathway with siRNA or small molecule inhibitors can sensitize cancer cells to TRAIL (3,20,28,38). The efficacy of PI3K/Akt/mTOR pathway inhibitors in combination with TRAIL has been previously demonstrated (28,39,40), while the effect of BKM120 and TRAIL co-treatment has not been examined in glioma.

In our study, both the extrinsic and intrinsic pathways of cellular death are implicated. As monotherapy, neither TRAIL nor BKM120 demonstrated a robust change in the mitochondrial Δψm, while the combination induced loss of mitochondrial Δψm. Moreover, analysis of the cytosolic fraction demonstrated the presence of Smac/DIABLO, AIF and cytochrome c, all critical players in the intrinsic pathway. Regulation of the mitochondrial pathway occurs via an intricate balance between expression levels of pro-apoptotic and pro-survival members of the Bcl-2 family, and levels of two proteins in this family, Mcl-1 and Noxa, appear critical.

It is well-established that Mcl-1, a known downstream regulator of the PI3K/Akt pathway, plays a prominent role in the inhibition of apoptosis, while Noxa is crucial in facilitating apoptosis; cellular death following co-treatment may be more contingent on levels of Noxa than Mcl-1. In this regard, we observed significant upregulation of the pro-apoptotic mediator Noxa associated with sequestration of the anti-apoptotic mediator Mcl-1, without significant changes in other Bcl-2 family members, suggesting the important role of these targets in regulating the responses observed. While it has been reported that TRAIL actually increases levels of the pro-survival protein Mcl-1, likely contributing to TRAIL resistance, studies have shown that a second agent that can reduce Mcl-1 levels could serve as effective co-treatment (14).

After knock-down of Mcl-1, we did not observe a statistically significant increase in the amount of cell death from control. On the other hand, western blot analysis showed an increase in the levels of Noxa after treatment with BKM120 alone and co-treatment. As shown by immunoprecipitation, Noxa sequestered Mcl-1, suggesting that levels of the pro-apoptotic protein Noxa may be more critical to the mechanism of cellular death than Mcl-1. shRNA experiments demonstrated that the loss of expression of Noxa limited the ability of this combination to produce cell death. In short, Noxa upregulation may post-transcriptionally inactivate Mcl-1 and allow for displacement of other pro-apoptotic proteins bound to Mcl-1 (41,42).

Our study showed an increase in total death receptor protein levels and death receptor expression at the cellular membrane, specifically DR5 and not DR4, after treatment with BKM120. Since TRAIL triggers apoptosis by binding DR4 and DR5, the expression of death receptors may be pivotal in determining the apoptotic response to death receptor-mediated cell death (43). Multiple recent studies have shown that the upregulation of death receptors, in particular DR5, by other agents such as proteosome inhibitors (44), amiodorone (45), arsenic trioxide (46), and platinum-based chemotherapies (47), can sensitize TRAIL-resistant glioma cell lines to TRAIL-induced cellular death and the use of sensitizers that can upregulate DR5 expression may allow for the selective death of glioma, sparing normal human astrocytes. Recently, an intimate connection has been shown to exist between diminished Akt pathway activation and an increase in DR5 expression (48). Nonetheless, a study evaluating co-treatment with BKM120 and TRAIL in lung cancer cell lines, at the same doses as used in our study, examined levels of DR4 and DR5 after treatment with BKM120 for 16 h and saw no change in receptor levels (in fact, a decrease in DR4 levels) (28), in contrast to the clear increase in DR5 expression we observed in human glioma. The mechanism by which PI3K/Akt pathway inhibition may lead to DR5 upregulation remains to be elucidated. The effect of PI3K/Akt pathway inhibition on the expression of death receptors deserves further examination and future studies looking at this combination should consider evaluation of extrinsic pathway proteins.

This study demonstrates that BKM120 augments TRAIL-induced apoptosis in proven TRAIL-resistant glioma cell lines. The combination shows efficacy in primary patient samples and no evidence of significant cellular death in normal human astrocytes. While Mcl-1 is downregulated by combination treatment, it appears that increased levels of Noxa are critical to cellular death in glioma. Moreover, treatment of TRAIL-resistant glioma cells with BKM120 increases expression of DR5, but not DR4, in both the intracellular milieu and at the cell surface. Our findings suggest further investigation of this combination *in vivo* is warranted.
